# Shaking-B misexpression increases the formation of gap junctions but not chemical synapses between auditory sensory neurons and the giant fiber of *Drosophila melanogaster*

**DOI:** 10.1371/journal.pone.0198710

**Published:** 2018-08-17

**Authors:** Sami H. Jezzini, Amelia Merced, Jonathan M. Blagburn

**Affiliations:** Institute of Neurobiology, University of Puerto Rico Medical Sciences Campus, San Juan, Puerto Rico, United States of America; EPFL, SWITZERLAND

## Abstract

The synapse between auditory Johnston’s Organ neurons (JONs) and the giant fiber (GF) of *Drosophila* is structurally mixed, being composed of cholinergic chemical synapses and Neurobiotin- (NB) permeable gap junctions, which consist of the innexin Shaking-B (ShakB). Previous observations showed that misexpression of one ShakB isoform, ShakB(N+16), in a subset of JONs that do not normally form gap junctions results in their *de novo* dye coupling to the GF. Misexpression of the transcription factor Engrailed (En) in these neurons also has this effect, and in addition causes the formation of new chemical synapses. These results, along with earlier studies suggesting that gap junctions are required for the development of some chemical synapses, led to the hypothesis that ShakB would, like En, have an instructive effect on the distribution of mixed chemical/electrical contacts. To test this, we first confirmed quantitatively that ShakB(N+16) misexpression increased the dye-coupling of JONs with the GF, indicating the formation of ectopic gap junctions. Conversely, expression of the ‘incorrect’ isoform, ShakB(N), abolished dye coupling. Immunocytochemistry of the ShakB protein showed that ShakB(N+16) increased gap junctional plaques in JON axons but ShakB(N) did not. To test our hypothesis, fluorescently-labeled presynaptic active zone protein (Brp) was expressed in JONs and the changes in its distribution on the GF dendrites was assayed with confocal microscopy in animals with misexpression of ShakB(N+16), ShakB(N) or, as a positive control, En. Using different methods of image analysis, we confirmed our previous result that En misexpression increased the chemical synapses with the GF and the amount of GF medial dendrite branching. However, contrary to our hypothesis, misexpression of ShakB did not increase these parameters. Immunostaining showed no association between presynaptic active zones and the new ShakB plaques, further evidence against the hypothesis. We conclude that both subsets of JON form chemical synapses onto the GF dendrites but only one population forms gap junctions, comprised of ShakB(N+16). Misexpression of this isoform in all JONs does not instruct the formation of new mixed chemical/electrical synapses, but results in the insertion of new gap junctions, presumably at the sites of existing chemical synaptic contacts with the GF.

## Introduction

There are two types of synapse in the nervous system, chemical and electrical, which are molecularly and physiologically disparate. Chemical synapses involve an enormously complex array of presynaptic transmitter release mechanisms, postsynaptic receptors, and signaling pathways, whereas electrical synapses are comprised of apparently simple cylindrical clusters of gap junctional proteins. In the past it was thought that these simpler electrical synapses were more abundant in ‘lower’ vertebrates and invertebrates, and were of lesser importance in adult mammals. It is now clear, however, that this is not the case [1,2], and that these two synapse types are often found together, located in close proximity and interacting functionally, in the adult brain as well as during development [2].

**Fig 1:**
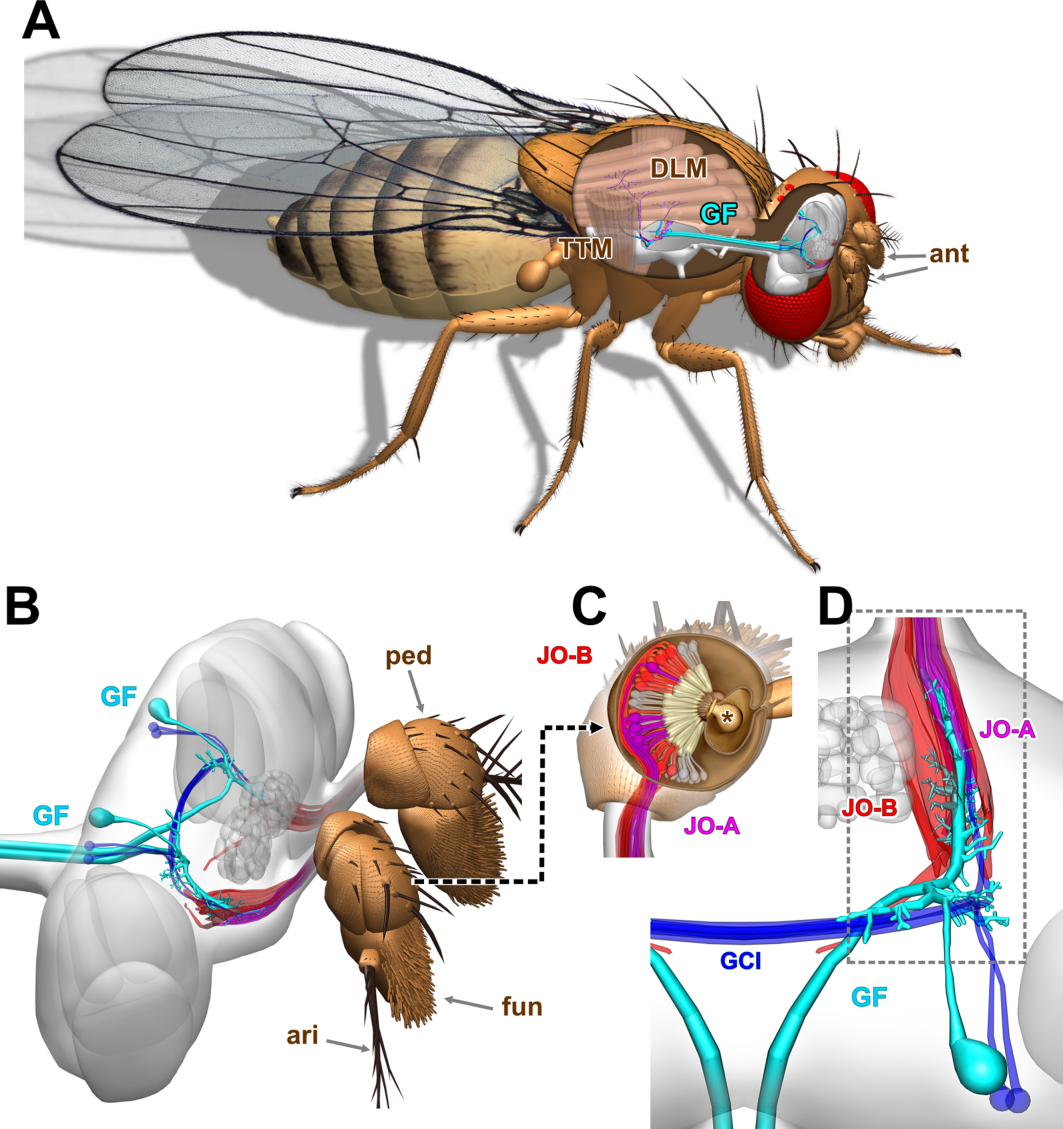
Johnston’s Organ inputs onto the *Drosophila* giant fiber escape circuit. (A) Overview of the giant fiber (GF) escape circuit, showing the GF axons descending from the brain to the thoracic ganglion, where they contact neurons that excite the tergotrochanteral ‘jump’ muscle (TTM) and the dorsal longitudinal indirect flight muscle (DLM). The antennae, one of the sources of input to the GF, are indicated (ant). (B) Side view of the pair of Giant Fiber (GF) neurons in the brain (cyan), showing their axons projecting posteriorly in the cervical connective to the thorax (left), and their main dendrites projecting anteriorly (right) to contact axons of Johnston’s Organ neurons (JONs) originating in the second antennal segment, or pedicel (ped). Two subsets of JONs are indicated here, the JO-A (red or magenta) and JO-B (red) sound-sensitive types. The third and fourth antennal segments, i.e. the funiculus (fun) and arista (ari), are also indicated. (C) Dorsal view of the right pedicel with the top removed to show the JONs within, with their dendrites attached to the funicular stalk (asterisk). Two subpopulations of neurons are shown, the JO-A (magenta) and JO-B JONs (red). (D) Dorsal view of the right GF neuron, also showing the neighboring GCI neurons (dark blue). The main GF dendrite projects anteriorly within a cylindrical group of JO-A axons, which are dye-coupled to it. The dashed box indicates the region shown in subsequent figures.

These ‘mixed’ chemical and electrical synapses are a typical feature of escape circuits in vertebrates and invertebrates [2], where the gap junctions are formed from connexin proteins, or the functionally analogous innexins, respectively [3]. In *Drosophila melanogaster* 8 genes for innexins have been identified [4]. One of the most thoroughly studied of these proteins, Shaking-B (ShakB), is a critical component of the giant fiber (GF) portion of the escape circuitry [5–7] (Fig 1). ShakB-containing gap junctions are present, along with cholinergic chemical synapses [8], at contacts made by the GF with two of its output neurons, allowing the fast activation of the ‘jump’ tergotrochanteral muscle (TTM) followed shortly by the dorsal longitudinal flight muscle (DLM) [9] (Fig 1).

The GF responds to a synchronous combination of visual stimuli (ie. rapid looming) [7,10], and air movements [11] detected by the neurons of the antennal Johnston’s Organ (JO), the *Drosophila* analog of the mammalian inner ear [12]. There is strong evidence that ShakB is also required for these excitatory inputs onto the dendrites of the GF: the *shakB*^*2*^ mutation disrupts visual circuitry [13], and abolishes synaptic currents from auditory neurons in the JO [14]. We recently showed that, despite the presence of chemical contacts, transmission of the response to sound at the JO neuron (JON)–to–GF synapses is indeed primarily electrical [15] and that ShakB is also the most likely component of these electrical synapses onto the main dendrite of the GF [16].

The idea that gap junctions are involved in determining the development of synaptic connectivity is not new; ironically ShakB itself was first postulated to be a synaptic recognition molecule [17]. There is experimental evidence for this phenomenon in several systems. For example, the innexins Ogre and ShakB(N) are required for the proper formation of the histaminergic synapses that connect *Drosophila* retinal and lamina neurons [13]. In the leech, gap junction expression between identified neurons is a prerequisite for normal chemical synapse formation [18]. In mammals, both synapse elimination by motoneurons, and the maturation of olfactory synapses, are disrupted in connexin mutants [19,20].

Our previous work suggested that gap junction proteins can also affect synaptic connectivity at the JON-to-GF synapse. In the innexin study we observed that overexpression of the ‘correct’ ShakB isoform, ShakB(N+16), in a subset of JONs induces ectopic synaptic coupling, whereas expression of the ‘wrong’ isoform, ShakB(N), abolishes it entirely [16]. In an earlier paper we had shown that ectopic synaptic coupling could also be induced by overexpression of the transcription factor Engrailed (En) in those same sensory neurons–in that case we showed that it was accompanied by the *de novo* formation of chemical synapses, along with an increase in postsynaptic dendritic branching [15]. Taken together, these results suggested the possibility that both En and ShakB(N+16) are similarly able to alter the specificity of the JON-GF synaptic connection, by causing the formation of new mixed electrical and chemical synapses between inappropriate synaptic partners. Here we test this idea experimentally by assaying dye coupling, the distribution of putative active zones on the GF dendrites, and the distribution of GF dendrites themselves, comparing overexpression of the two different ShakB isoforms to that of En. We find that, contrary to our original hypothesis, only En is able to alter the distribution of chemical synapses (and GF dendrites), while ShakB overexpression affects gap junctional coupling alone.

## Materials and methods

### Flies

*Drosophila melanogaster* flies of the following genotypes were obtained from the Bloomington Stock Center: *UAS-mCD8*::*GFP* (5130 or 5137), *JO15-GAL4* (6753). Other lines used were *UAS-shakB(N)* and *UAS-shakB(N+16)* {Pauline Phelan [6]}, *UAS-en* {Miki Fujioka [21]}, *UAS-brp-short-strawberry* {Stephan Sigrist [22]}. A GFP-tagged chromosome 2 balancer containing *CyO (*denoted *CyO-GFP* below) was obtained from Bruno Marie [23]. The following fly lines were constructed in the laboratory:

*UAS-brp-short-strawberry/ CyO-GFP; UAS-en*/*TM6B*, *Tb*^*1*^,
*UAS-brp-short-strawberry/ CyO-GFP; UAS-shakB(N+16)*/*TM6B*, *Tb*^*1*^,
*UAS-brp-short-strawberry/ CyO-GFP; UAS-shakB(N)*/*TM6B*, *Tb*^*1*^,
*UAS-mCD8*::*GFP/ CyO-GFP;JO15-GAL4/TM6B*, *Tb*^*1*^,

*GAL4* lines were crossed with the respective *UAS* lines and the F1 used for experiments. Flies were reared on cornmeal media and raised at 25°C and 60% relative humidity. Adults from 3–10 days old were used for experiments.

### Dye coupling

Dissection and dye injection were performed as previously described [15]. Briefly, animals were dissected so as to expose the cervical connective and reveal the GF axons. One of the GF axons was impaled with a sharp glass microelectrode, the tip of which was filled with a mixture of 3% Neurobiotin Tracer (NB) (Vector Labs, SP-1120) and 3% Dextran Alexa Fluor 488 (DA488) (10,000 MW, Bioanalytical Instruments, D22910) diluted in distilled water. Injection electrodes were backfilled with 150 mM potassium chloride and had resistances of 45–60 MΩ. The dye mixture was iontophoretically injected for up to 20 minutes using a continuous train of alternating 1 second square pulses of positive and negative current of ± 1–2 nA generated by a Master 8 stimulus generator (A.M.P.I., Israel) and delivered through an AxoClamp 2B amplifier (Molecular Devices LLC, CA, USA).

### Immunohistochemistry

The nervous systems from dye-injected flies were fixed in 4% paraformaldehyde in phosphate-buffered saline (PBS) for 45 minutes at 4°C, rinsed in several changes of PBS and further dissected to remove the brain and ventral nerve cord. To process other flies, nervous systems were dissected in PBS, fixed in 4% paraformaldehyde in PBS for 30 minutes at room temperature and rinsed in several changes of PBS. Samples were then processed for antibody labeling and cleared and mounted as previously described [15]. The nc82 (anti-Bruchpilot) monoclonal antibody was obtained from the Developmental Studies Hybridoma Bank (DSHB) and used at a dilution of 1/20. Rabbit polyclonal anti-ShakB [6] (J.P. Bacon, University of Sussex) was used at a dilution of 1/500. Goat anti-mouse secondary antibody labeled with Alexa-555, and goat anti-rabbit secondary antibody labeled with Pacific Blue (Thermofisher Scientific P-10994), were applied at 1/500 dilution. Streptavidin-conjugated Pacific Blue (Molecular Probes S11222) was used at 1/2000. Preparations were examined using either a Zeiss Pascal or Nikon Eclipse T1 A1r laser scanning confocal microscope and images were acquired at 8 bit resolution.

### Image processing and analysis

Confocal image stacks were imported into Fiji image analysis software [24,25], with which they were 3D-rotated to a standard position and adjusted for optimal contrast. The large artefactual Brp-sh-Strawberry agglomerations noted in [15] were automatically removed from the red channel by applying a Minimum filter (3 pixels) followed by a Maximum filter (3 pixels), subtracting the result from the original using the Image Calculator, then readjusting for optimal contrast. This procedure reliably removed the agglomerations, which do not stain with Brp antibody, without affecting the actual Brp-positive active zones (S1 Fig).

**Fig 2:**
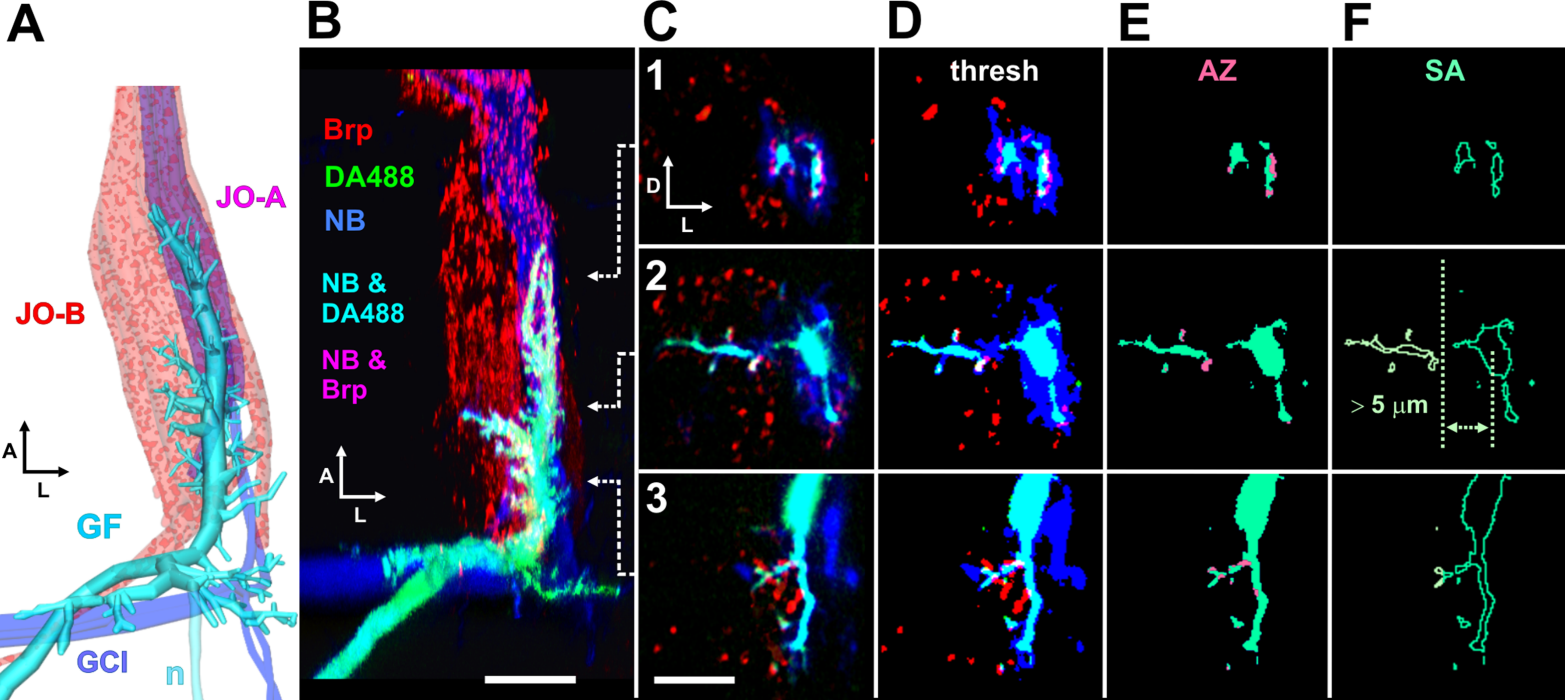
Quantification of GF synaptic inputs and branching. (A) Diagrammatic view of the GF dendrite (cyan) and JON axons (red spots), similar to that shown in the box in Fig 1D. The JO-A group of axons (magenta) is NB-coupled to the GF. The GCI neurons are also NB-coupled to the GF. The posterodorsally-projecting GF neurite (n) is cropped out of subsequent images. Anterior (A) is towards the top, lateral (L) to the right. (B-C). Confocal views of a typical preparation. The right GF axon was injected with a mixture of Neurobiotin (NB: blue) and Alexa Fluor 488 –coupled dextran (DA488: green), making it cyan in color. *JO15-GAL4* was used to drive expression of Strawberry-tagged Brp-short (Brp: red) in JO-A and JO-B subgroups of JON axons, labeling putative active zones at chemical synapses. Blue NB passes retrogradely into a subgroup of the JO-A axons, so their red active zones appear magenta, or white when apposed to the GF. (B) Dorsal 3D view of the complete image stack of frontal slices, with anterior (A) towards the top, lateral (L) to the right. The subsequent panels represent single frontal slices taken at the antero-posterior positions indicated by the arrows: 1, at the anterior end of the GF dendrite, 2, midway along the dendrite in the region of medial branches, and 3, at the posterior end of the dendrite, where it bends dorsally and extends a ventral branch. In these panels, dorsal is to the top and lateral to the right. (C) Single confocal slices taken at the indicated regions, with dorsal (D) towards the top, lateral (L) to the right. Regions of overlap of Brp signal with the cyan GF dendrites (ie. putative synaptic contacts) appear white. (D) Thresholded signal. (E) NB signal removed to show only putative active zones (AZ: pink) on the GF. The total AZ volume over the length of the GF dendrite was subsequently quantified. (F) Single pixel outline of the GF dendrites, used as an approximation for surface area. Dendrites projecting medially more than 5 um were masked (pale green) and quantified separately. Scale bars: 20 μm in A, 10 μm in B-E.

Image quantification was similar to that described previously [15]. Using Fiji, the channels were Autothresholded using the Intermodes method for Brp, the Default method for the DA488 and NB signals, and the RenyiEntropy method for the ShakB signal. In all cases, the Ignore white, Stack, and Stack Histogram options were selected. The ROI was restricted to the JON afferent axons within the brain and the primary GF dendrite by first trimming the image in the 3D viewer. The thresholded images were binarized and the Measure Stack macro was used to quantify the total area of signal per slice. Areas of overlap of the Brp signal with the GF dendrites (putative active zones) were obtained using the AND operation in the Image Calculator (Fig 2E). The unbiased Brp filtering method described above eliminated the spuriously large amount of overlap of Brp and GF that was otherwise encountered at the posterior region of some of the GF arbors (S1 Fig). The GF dendritic surface area was estimated by measuring the total volume of a single-pixel outline (obtained by subtracting a Minimum-1-pixel-filtered image from the thresholded original) (Fig 2F), then dividing by the pixel size (0.22 μm). Medially-projecting dendrites were selected by masking out all dendrites that fell within a boundary that was 5 μm (23 x 0.22 μm pixels) medial from the center point of the GF dendrite (Fig 2F). For the ShakB versus Brp staining, the GF dendrite was not visible so GFP-labeled JON axons were divided into A and B subpopulations by visual tracing and manual masking, followed by subtraction from the original image. The degree of retrograde Neurobiotin (NB) coupling from GF to JONs was measured by the mean cross-sectional area of NB signal averaged from 10 x1 μm slices centered on the anteriormost tip of the GF dendrite.

Maximum intensity Z-series projections created in ImageJ were imported into Adobe Photoshop for construction of figure plates. The Fluorender program was used for the 3D views [26]. Blender was used for the construction of the diagrams in Fig 1. The GF was traced in Neutube [27] and saved as swc file. This was converted to vtk format using a Python script (swc2vtk: Daisuke Miyamoto) and then to x3d format in ParaView (paraview.org). The x3d file was then imported into Blender (blender.org) for final edits and rendering. The other 3D structures of the fly body and CNS were modeled in Blender, rendered, then layered in Photoshop. Final graphics for all figures were composed and labeled using CorelDraw.

### Statistics

N represents the number of animals. The normality of the distribution of the data sets was first determined then subsequent tests were carried out using PAST3 software [28]. To identify significant differences between means of control vs. experimental groups, normally-distributed data were compared with ANOVA followed by *post-hoc* Tukey tests. In figures, * denotes p ≤ 0.05, ** p ≤ 0.01, *** p ≤ 0.001. Plots were made with Excel and transferred to CorelDraw for construction of the graphs.

## Results and discussion

The Johnston’s Organ (JO) in the antennal second segment, or pedicel, is a large chordotonal organ containing around 480 sensory neurons [12,29]. Some of these JO neurons, or JONs, detect sound (JO-A and B subtypes: Fig 1), while another subpopulation responds to gravity and wind (JO-C and E) [14,30–32]. The *JO15-GAL4* line [33] is expressed strongly in all of the JO-A and the majority of the JO–B neurons [29] (Figs 1C, 1D and 2A). We have shown previously that normally only a subset of the JO-A neurons, those which express the transcription factor En, are NB-coupled to the GF [15]. Using *JO15-GAL4* to drive misexpression of En in both JO-A and JO-B JONs causes the axons of the latter to become NB-coupled to the GF, and also results in the formation of new chemical synaptic connections between these ‘inappropriate’ synaptic partners, along with a concomitant increase in the length of medial GF dendrites [15]. Later, qualitative, observations suggested that, despite its apparent dissimilarity in molecular structure and function, overexpression of the N+16 isoform of ShakB also had an identical effect on dye coupling [16]. Our hypothesis, therefore, is that overexpression of ShakB(N+16) will also cause the formation of new, mixed, synaptic connections between JON afferents and GF, resulting in the appearance of more putative active zones in contact with the GF dendrites in addition to the NB-coupling of more axons.

### ShakB(N+16) increases NB coupling while ShakB(N) abolishes it

**Fig 3:**
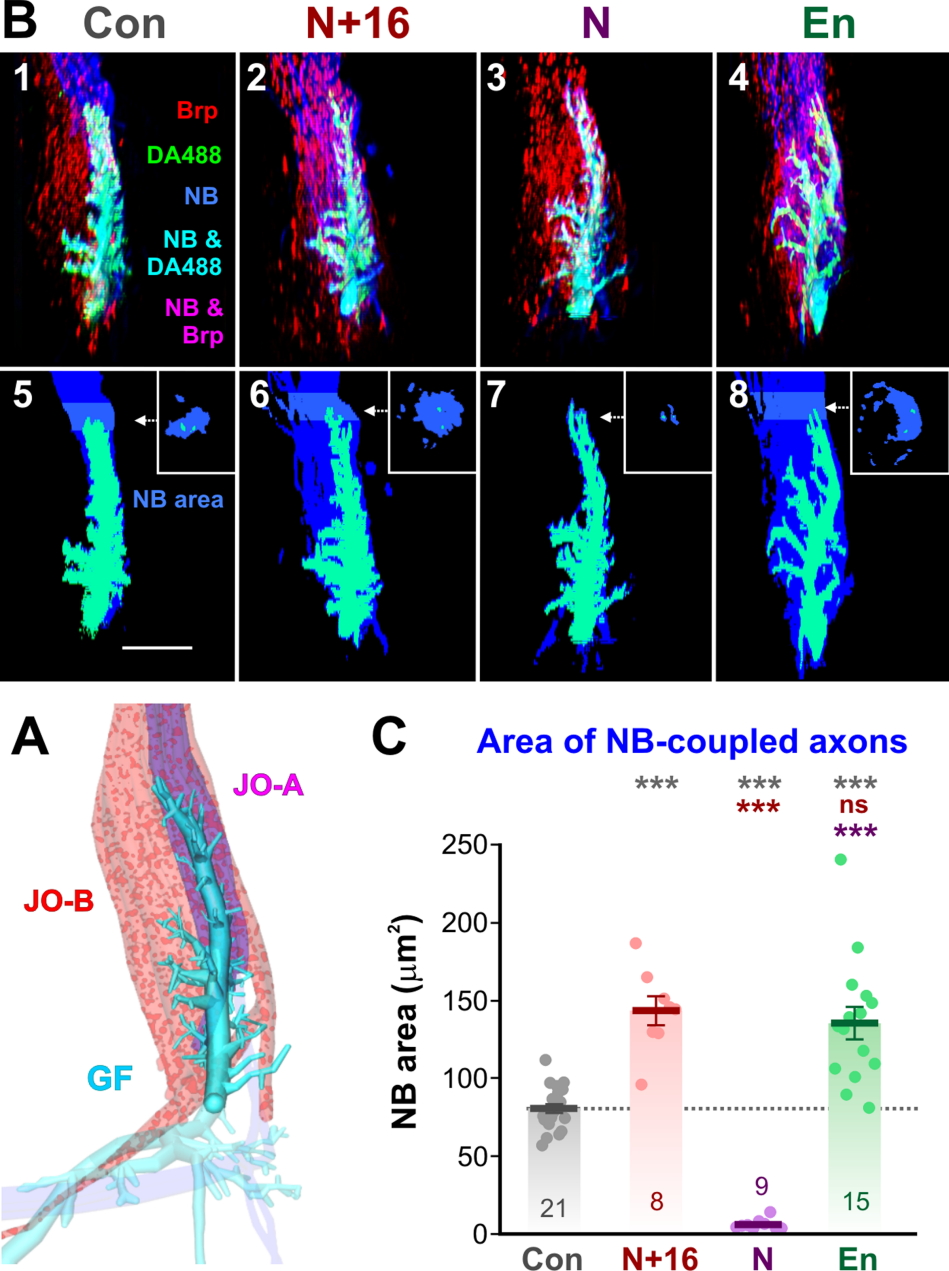
ShakB(N+16) increases, and ShakB(N) decreases, NB coupling. (A) Diagrammatic dorsal view of the same GF shown in Fig 1D, showing only the analyzed portion of the main dendrite. JO-A and JO-B axons are labeled with red Brp spots, while JO-A axons are also magenta indicating NB-coupling. (B) Examples of experimental preparations, showing dorsal views of cropped image stacks of the GF dendrite and JON axons, with anterior towards the top, lateral to the right. The analyzed portion of the right GF dendrite is shown (cyan), along with Brp labeling in JON axons (red) and NB coupling (blue). Panels 1–4 show control animals (Con, column 1), *UAS-shakB(N+16)* animals (N+16, column 2), *UAS-shakB(N)* (N, column 3), and, as a positive control, *UAS-en* (En, column 4). Panels 5–8 show the corresponding GF and NB channels only and indicate the 10 μm-thick region at the tip of the dendrite from which the area of NB coupling was averaged. (C) Cross-sectional area of NB-coupled axons. Scatter plots combined with bar charts showing mean ± SEM. Asterisks or “ns” above each column indicate the significance when compared to preceding columns with *post-hoc* Tukey tests. Expression of N+16 caused a significant increase in coupling area, similar to that caused by En, whereas the ShakB(N) isoform abolishes NB coupling.

We assessed changes in dye coupling by injecting the GF axon with a mixture of the dyes Dextran Alexa Fluor 488 (DA488) and Neurobiotin (NB). We used the former instead of the previously used Lucifer Yellow [15,16] because LY passes retrogradely thorough the new gap junctions induced by ShakB(N+16), making it difficult to distinguish the GF dendrites from coupled axons [16]. The *JO15-GAL4* line was used to drive *UAS-brp-sh-strawberry* in the JO-A and JO-B populations of mechanosensory afferents, as before, in order to label putative presynaptic active zones (AZ) [22]. The cross-sectional area of NB-coupled axons was averaged from 10 x 1 μm slices taken at the anterior tip of the GF dendrite, where the axons leave the antennal nerve and enter the neuropil of the antennal mechanosensory and motor center (AMMC) (Fig 3B5–3B8). As a positive control we reanalyzed previous En misexpression preparations using our new methods of image filtering and data analysis.

There is a clear 77% increase in the cross-sectional area of NB coupling with ShakB(N+16) overexpression (Fig 3B6 and 3C). In comparison, this is not significantly different from the 68% increase in NB coupling area seen in *UAS-en* animals (Fig 3B8 and 3C), and clearly results from the *de novo* dye coupling of a new population of JON axons, presumably the same JO-B axons in both cases. Conversely, expression of the ShakB(N) isoform completely abolishes the normal dye coupling of the JO-A axons (Fig 3B7 and 3C). Presynaptic ShakB(N+16) is required for a functional JO-A-to-GF synapse [16], and it is the only isoform known to be expressed by the postsynaptic GF [6]. Our results quantitatively confirm those of the previous study [16], and show that misexpression of ShakB(N+16) in JONs induces the formation of NB-coupling, presumably homotypic, gap junctions between JO-B axons and GF, while expression of the non-functional Shakb(N) isoform removes or inhibits the existing junctions between JO-A axons and GF. Whether the electrical coupling is similarly affected remains to be tested in a future electrophysiological study.

### ShakB(N+16) expression increases ShakB immunostaining in JO axons

**Fig 4:**
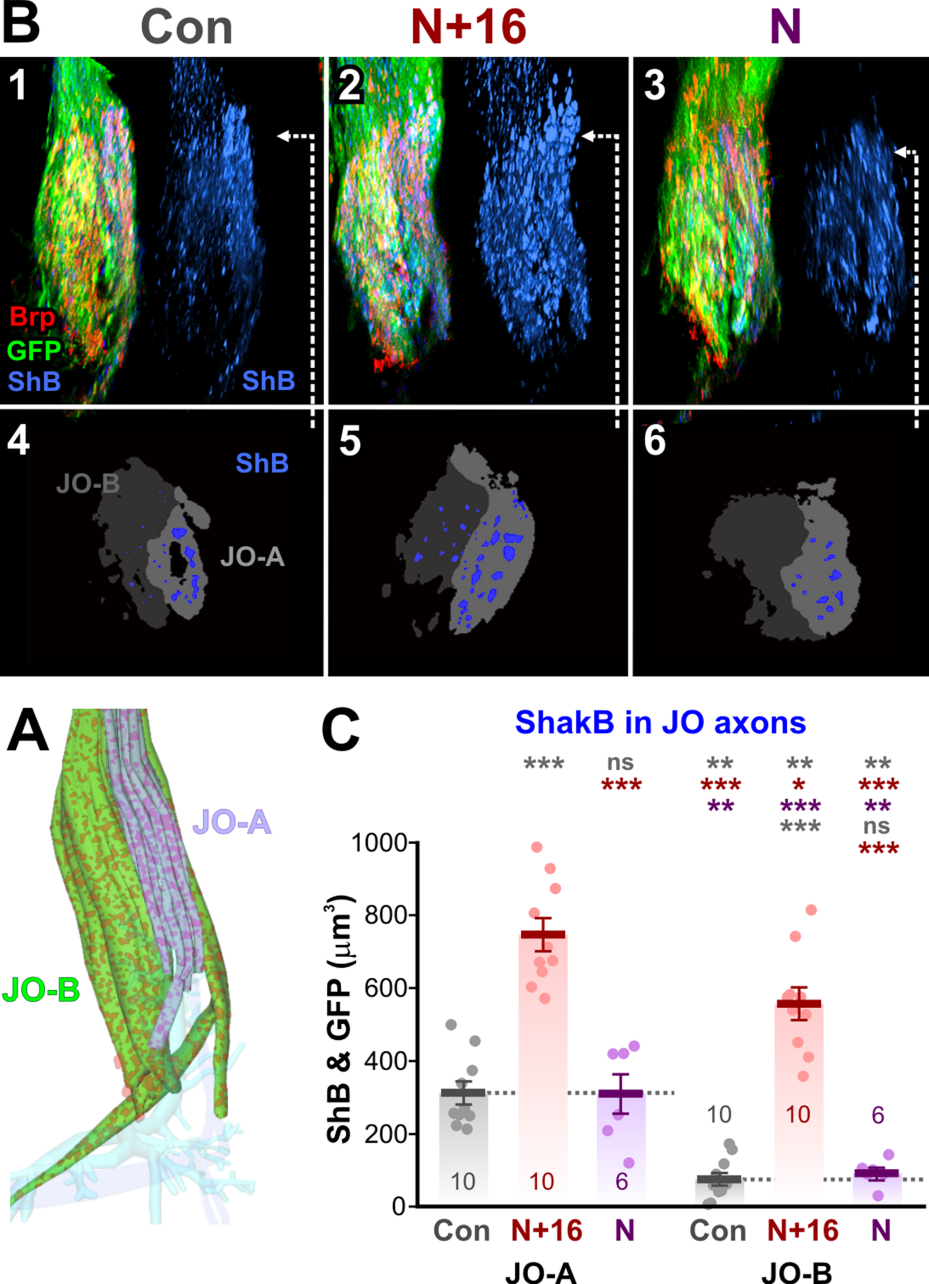
ShakB immunostaining is increased by ShakB(N+16) overexpression. (A) Diagrammatic dorsal view of the same region of JON afferents shown in previous figures. Both JO-A and JO-B axons are labeled with green GFP and red Brp spots, while the cylindrical cluster of JO-A axons is highlighted for clarity (lilac). (B) Typical preparations, in which *JO15-GAL4* was used to drive *UAS-brp-sh-strawberry* (Brp) and also *UAS-CD8*::*GFP* (GFP) in the JO-A and JO-B neurons. The GF was not labeled. ShakB protein was stained with antibody against all isoforms (ShB). Panels 1–3 show examples of experimental preparations, showing dorsal views of cropped image stacks of the JON axons, with anterior towards the top, lateral to the right. To the right of each is the ShB channel alone. Control animal (Con, column 1), *UAS-shakB(N+16)* animal (N+16, column 2), and *UAS-shakB(N)* (N, column 3). Arrows indicate the approximate position of the single frontal slices shown in subsequent panels. (B4-6) Single thresholded frontal slices, dorsal towards the top, lateral to the right. Plaques of ShB labeling (blue) are shown in the two subsets of axons, JO-A (light gray) and JO-B (dark gray). (C) Total volume of ShakB staining in the two subsets of JO axons. Scatter plots combined with bar charts showing mean ± SEM. Asterisks or “ns” above each column indicate the significance when compared to preceding columns with *post-hoc* Tukey tests. Control JO-A axons have more ShakB staining than JO-B. The N+16 isoform increases ShakB staining in both JO-A and JO-B axons, while the N isoform does not.

For these experiments the GF was not dye-filled; instead we labeled the JO-A and JO-B axons themselves by driving *UAS-CD8*::*GFP* expression along with *UAS-brp-sh-strawberry* (Fig 4). The preparations were processed for immunostaining with an antibody that recognizes all known isoforms of the ShakB protein [6]. We directly traced the JO-A and JO-B axons based on the known anatomy of their arborizations [29], and separate masks were made for each in order to quantify the localization of ShakB staining. ShakB immunoreactivity is typically concentrated in plaques of approximately 1 μm diameter [9,16], and in controls these plaques are visible in the region of the JO-A axons that cluster around the GF dendrite (Fig 4B1 and 4B4).

In controls, there is approximately 4-fold more ShakB immunostaining in the region of the JO-A axons than in the JO-B axons (Fig 4B4 and 4C). This correlates with the normal lack of dye coupling of the GF with the JO-B axons. With expression of *UAS-shakB(N+16)* in both JO-A and JO-B neurons, the amount of ShakB immunostaining is greatly increased in both populations (Fig 4B5 and 4C). This is consistent with the formation of new gap junctional plaques that allow increased dye coupling to these axons, in accordance with previous evidence that it is ShakB(N+16) that is the functional component of the JON-GF electrical synapses [16].

Conversely, no change in ShakB immunoreactivity is seen when *UAS-shakB(N)* is expressed (Fig 4B6 and 4C). This indirectly confirms that the increase in staining seen with ShakB(N+16) expression is indeed due to the appearance of new gap junctional plaques and not simply to an non-specific accumulation of the protein in the axons–if the latter were the case we would expect an increase with ShakB(N) expression as well since the antibody recognizes both. This result also suggests that the ShakB(N) isoform, which is apparently not required for the functioning of the synapse [16], is unable to promote the assembly of additional gap junctional plaques. It does not, however, appear to reduce ShakB staining below control levels, and therefore does not completely inhibit the formation of gap junctions; those remaining presumably include the native ShakB(N+16) protein. However, ShakB(N) does have a dominant negative effect on NB-coupling, completely preventing it even though ShakB plaques remain. The sequences of ShakB(N) and (N+16) are identical except for the eponymous 16 amino-acids on the N-terminus of the latter, which is intracellular [34]. The N-terminus of ShakB is known to be important for voltage sensitivity, and also probably for trafficking and oligomerization [35]. One possibility is the presynaptic formation of heteromeric connexons composed of native ShakB(N+16) and ectopic ShakB(N), which stain with the ShakB antibody but, when docked with their homomeric ShakB(N+16) counterparts in the GF membrane [6], have an altered configuration that prevents dye coupling.

### ShakB(N+16) does not change the number of chemical synapses onto GF

**Fig 5:**
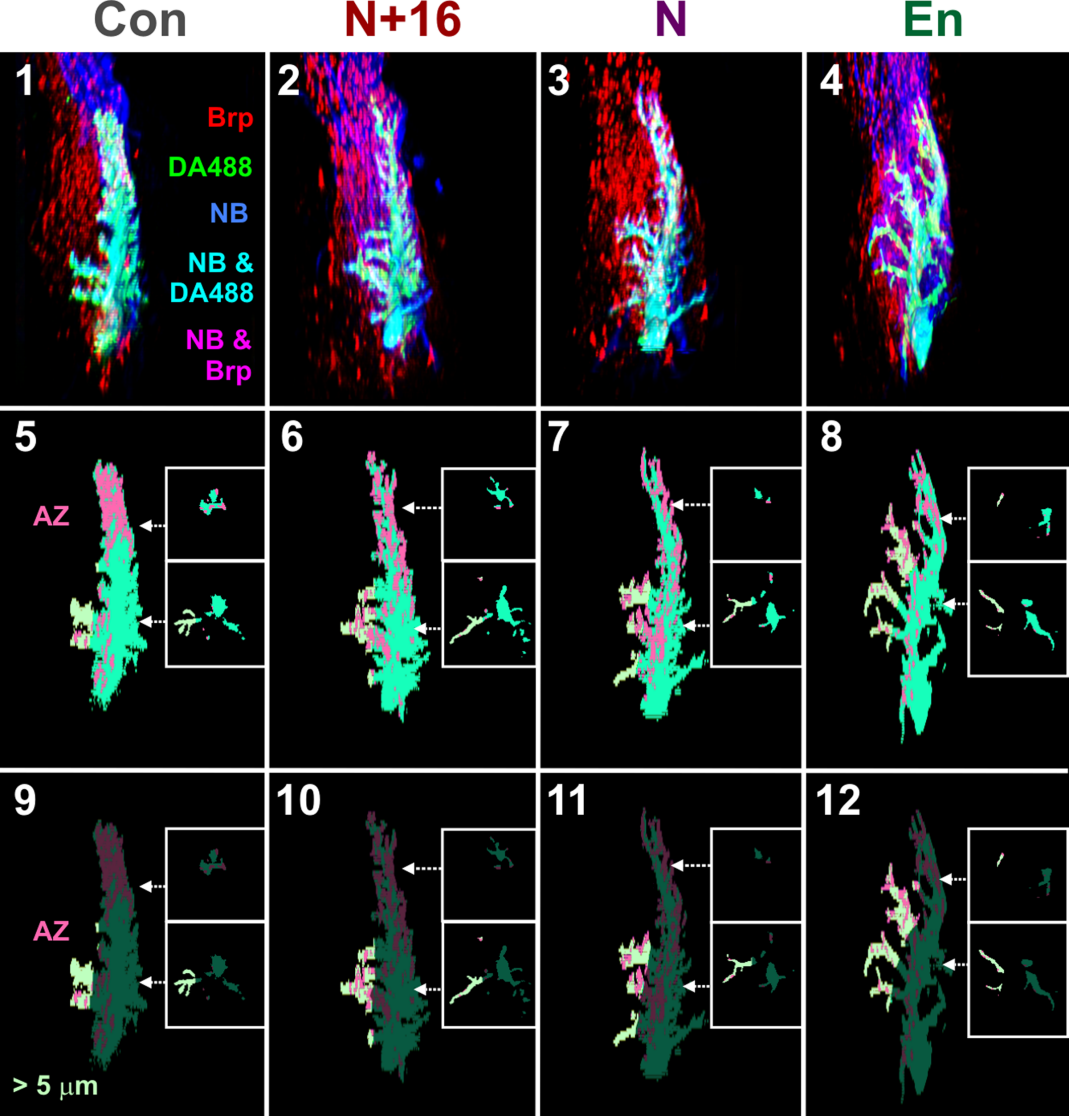
ShakB misexpression and active zone distribution on GF. Examples of experimental preparations, showing dorsal views of cropped image stacks of the GF dendrite and JON axons, with anterior towards the top, lateral to the right. The analyzed portion of the right GF dendrite is shown (cyan), along with Brp labeling in JON axons (red) and NB coupling (blue). Panels 1–4 show control animals (Con, column 1), *UAS-shakB(N+16)* animals (N+16, column 2), *UAS-shakB(N)* (N, column 3), and *UAS-en* (En, column 4). Panels 5–8 show the GF and Brp appositions (putative AZ) on the GF dendrites, with insets showing representative single frontal slices taken at the indicated antero-posterior positions. Medial GF dendrites are indicated by a pale green color. Panels 9–12 show putative AZ on only the medial GF branches (pale green).

**Fig 6:**
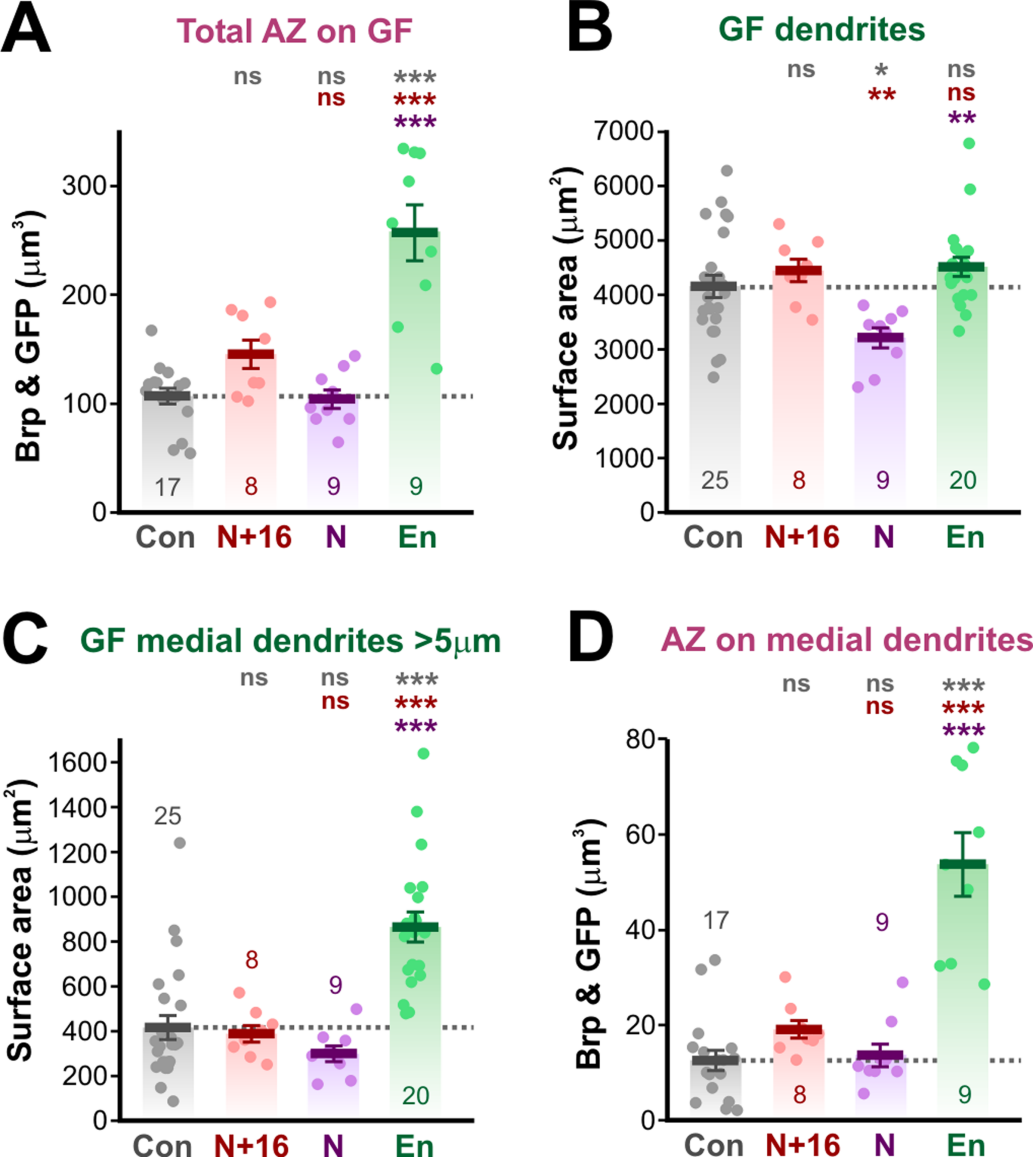
Quantification of effects of ShakB misexpression on chemical synapses and dendritic branches of GF. (A-D) Scatter plots combined with bar charts showing mean ± SEM. Asterisks or “ns” above each column indicate the significance when compared to preceding columns with *post-hoc* Tukey tests. (A) Total volume of AZ apposed to the GF. Neither ShakB(N+16) nor ShakB(N) affected the amount of AZ, compared to the increase seen with En overexpression. (B) Total surface area of GF dendrites. There is no effect of ShakB(N+16) or En misexpression but ShakB(N) causes a 23% decrease in branching. (C) Surface area of GF dendrites that project more than 5μm medially. There is no effect of ShakB(N+16) or ShakB(N) misexpression but En results in an increase in branching. (D) Total volume of AZ apposed to the medial GF dendrites. There is no effect of ShakB misexpression but En results in a large increase in medial branch AZ.

We have shown that overexpression of ShakB(N+16) and ShakB(N) affect NB coupling with JON axons in opposite ways, and that the former induces the formation of new gap junctional plaques. The main test of our hypothesis is to determine whether the amount of presynaptic active zones (AZ) at chemical synapses is similarly affected. Again, overexpression of En was used as a positive control, where we know that new putative chemical synapses are formed [15]. We estimated the numbers of putative AZ apposed to the GF dendrites by measuring the total volume of overlap of the Brp-sh and the DA488 signals. Neither the misexpression of ShakB(N+16) nor that of ShakB(N) has any significant effect on total AZ volume (Figs 5 and 6A), evidence against our initial hypothesis. In contrast, En does cause a 140% increase in putative AZ (Figs 5 and 6A), a result confirming our previous study that used a slightly different method of image processing and analysis [15].

ShakB(N+16) has no effect on total dendritic branch area, although ShakB(N) decreases it by 23% (Fig 6B). This decrease is perhaps a reaction by the GF to the loss of functional (as indicated by NB-coupling) gap junctions with the JO-A afferents. Neither ShakB isoform has any effect on the surface area of medial dendrites, which would be expected to disproportionately bear any new synapses because the JO-B axons are medially located (6C). In contrast, and in agreement with our previous study [15], En misexpression causes a large increase in medial dendritic branching (of 108%: Fig 6C) and an even larger (327%) increase in the AZ on those dendrites (Fig 6D).

### ShakB(N+16) increases ShakB colocalization with Brp

The existence of mixed synapses at the JON-GF connection implies that the ShakB gap junction protein and the Brp AZ protein should colocalize, at least within the limits of resolution of the confocal microscope. Electron microscopy of the mixed output synapses of the GF in the thoracic ganglion showed that AZ and the distinctive large gap junctions are interspersed in close proximity, often within 0.5 μm of each other [9]. Comparable high-resolution electron microscopic data on the JON-GF connection are not available, however, examination of open-access electron microscope serial sections of the adult brain [36] show that AZ and nearby areas of close membrane apposition (a prerequisite for the existence of gap junctions) are also common between the morphologically identifiable JO-A axons and the GF dendrite (S2 Fig). However, gap junctions *per se* are not clearly visible, perhaps because the 4 nm per pixel resolution of these images is too low.

**Fig 7:**
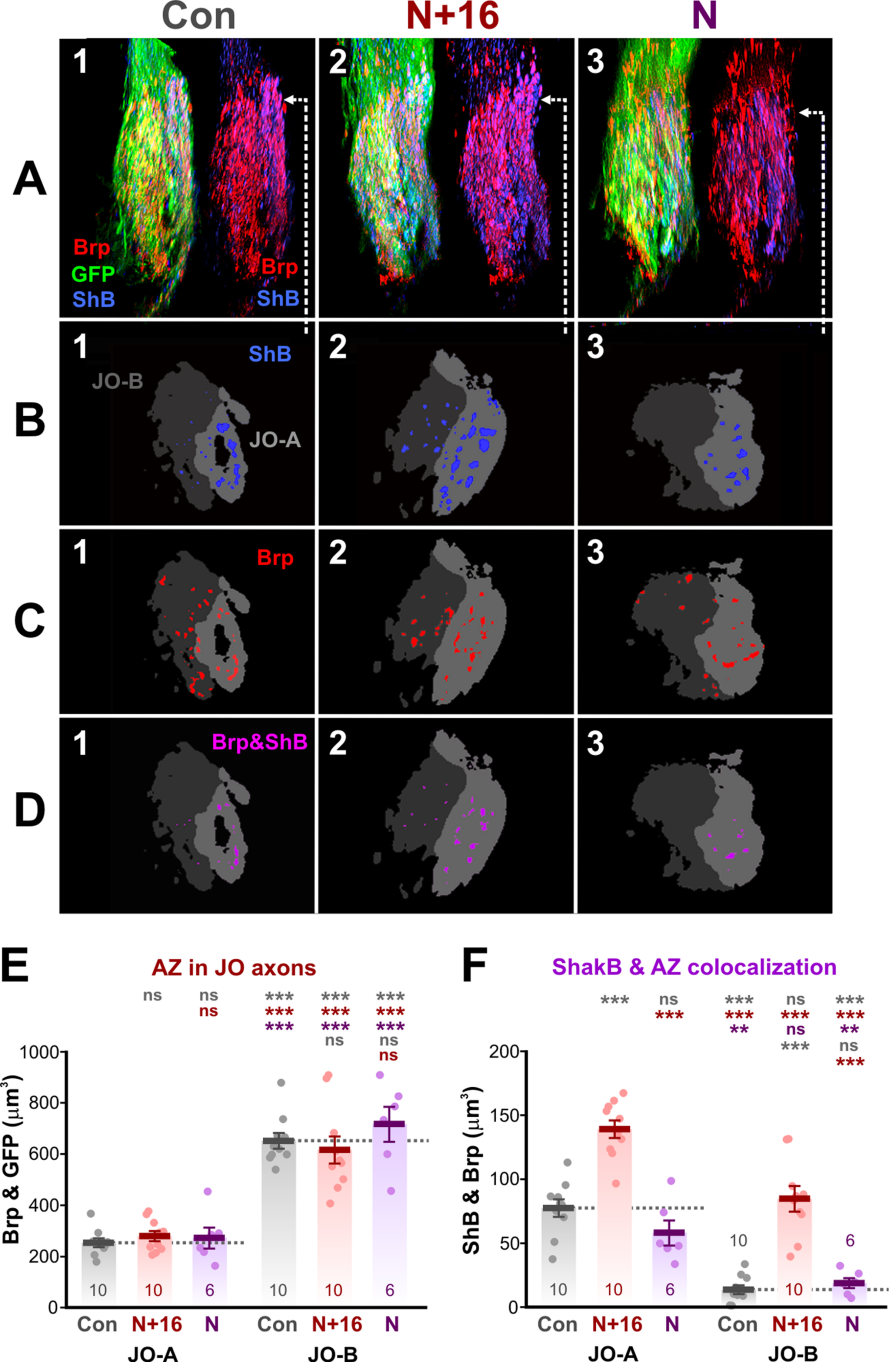
ShakB and AZ colocalization. (A) Typical preparations, in which *JO15-GAL4* was used to drive *UAS-brp-sh-strawberry* (Brp) and also *UAS-CD8*::*GFP* (GFP) in the JO-A and JO-B neurons. The GF was not labeled. ShakB protein was stained with antibody against all isoforms (ShB). Panels A1-3 show examples of experimental preparations, showing dorsal views of cropped image stacks of the JON axons, with anterior towards the top, lateral to the right. To the right of each the GFP channel is omitted to show the overlap between Brp and ShB. Control animal (Con, column 1), *UAS-shakB(N+16)* animal (N+16, column 2), and *UAS-shakB(N)* (N, column 3). Arrows indicate the approximate position of the single frontal slices shown in subsequent panels. (B) Plaques of ShB labeling in the two subsets of axons, JO-A (light gray) and JO-B (dark gray). (C) Putative AZ (Brp) in the JO-A and JO-B axons. (D) Overlap of Brp and ShB in the JO-A and JO-B axons. (E-F) Scatter plots combined with bar charts showing mean ± SEM. Asterisks or “ns” above each column indicate the significance when compared to preceding columns with *post-hoc* Tukey tests. (E) Total volume of putative AZ in the JO axons. More are present in JO-B than JO-A but ShakB isoform expression has no effect on either. (F) Colocalization of ShakB and AZ is significantly increased by the N+16 isoform in both A and B JO axons.

Puncta of Brp-sh fluorescence (putative AZ) are present in both the regions of JO-A and JO-B axons (Fig 7C1). This suggests that the JO-B axons form chemical synapses but does not necessarily mean that these are with the GF itself. However, as shown above (Fig 5), dendritic branches of GF which project too far medially to be in the region of JO-A axons do bear Brp puncta, suggesting that the JO-B axons can form chemical synapses with GF in the absence of gap junctions. In the JO-A axons however, Brp puncta tend to colocalize with ShakB (Fig 7D1). Taken together these results suggest that the JON-GF synapse is not obligatorily mixed but can consist of either gap junctions with chemical contacts or chemical contacts alone.

As a second test of the hypothesized determinative effect of ShakB on chemical synaptic connections, we assayed for co-localization of the ShakB protein with Brp puncta. Overexpression of ShakB, of either isoform, has no effect on the amount of Brp-sh labeling (putative AZ) in the JO axons (Fig 7C2, 7C3 and 7F). This is an independent confirmation of the results of Fig 6, which disprove our original hypothesis. Colocalization of AZ and ShakB increases with ShakB(N+16) expression (Fig 7F), but the percentage of ShakB that is colocalized with AZ remains constant at about 20–25% for JO-A and 15–20% for JO-B (not illustrated). Thus the likely explanation is that colocalization becomes more frequent simply because more ShakB plaques are present, not because there is some instructive link between insertion of gap junctions and the formation of chemical synapses.

### No evidence for instructive role of ShakB in the formation of chemical synapses

The results of this study disprove our initial hypothesis–we find no evidence that manipulating the numbers of functional gap junctions (as measured by dye coupling and by ShakB immunostaining) has any effect on the distribution of chemical synaptic connections (as measured by putative active zones). This cannot be due to the timing of expression of the *JO15-GAL4* driver because En, driven by the same driver, does indeed increase chemical contacts. This is perhaps not an unexpected result because, even in *shakB*^*2*^ mutants, in which ShakB(N+16) and ShakB(N) are absent, the chemical portion of the GF-TTMn synapse is still present [9], and is able to partially compensate functionally [8,37]. Thus, we conclude that JO-A neurons already express ShakB(N+16) and so form gap junctions alongside their chemical counterparts, but JO-B axons only do so when made to express ShakB(N+16) artificially. Thus, misexpression of ShakB(N+16) in JON-B axons simply results in the insertion of new gap junctions alongside chemical synapses that are already present between JO-B and GF.

The physiological function of the chemical synapses between JO axons and GF is obscure, because it has previously been shown that blocking them with Cd^2+^ or tetanus toxin has no apparent effect on either the postsynaptic currents in GF or its response to sound [14,15]. Morphological synapses with no discernible physiological function (ie. “silent synapses”) are in fact a common phenomenon in all nervous systems [38]. At the *Drosophila* neuromuscular junction conditionally silent Ib-type synaptic sites are important in short-term plasticity [39,40]; additionally, synaptic sites with no evoked release can evince miniature neurotransmission, which is particularly important during development [41,42]. It is a possibility that the JON-GF chemical synapses fulfil one or both of these functions. Finally, our conclusion that JON axons can form chemical presynapses with GF dendrites that have no discernible physiological function has a cautionary relevance for connectomics studies that are based on ultrastructure alone.

## Supporting information

S1 Fig Image filtering to remove artifactual Brp-sh agglomerations. The A column shows unfiltered images, the B column shows images with the red channel filtered to remove large agglomerations. Panels 1–3 show a preparation in which *JO15-GAL4* was used to drive UAS-brp-sh-strawberry (red: Brp-sh) and also UAS-CD8::GFP (green: GFP) in the A and B JONs. Native Brp protein was stained with nc82 antibody (blue: Brp). Panel1 is a maximum intensity Z-series projection through the length of the JON afferents, panels 2 and 3 are single slice views taken at the anterior tip and midway along the arbors, respectively. In these the GFP channel is removed for clarity. (A) Unfiltered images, showing large blobs that do not stain with nc82 (asterisks), some of which are associated with JO15-labeled cell bodies or axons. (B) Filtered images showing the almost complete removal of the artefactual blobs, leaving the nc82-stained Brp-sh puncta unaffected (arrowheads in 2 and 3). In panels 4 and 5 are shown preparations in which the GF axon was injected with NB (blue) and DA488 (green), making it cyan in color. Single sections are taken from the posterior region of the GF arbor. Panel 4 shows the same preparation and section as in Fig 2C3 –in this case filtering out the large blobs has little effect on the amount of overlap of Brp with the GF (white regions), although one contact region is greatly reduced (yellow arrow). In contrast, panel 5 shows the same region of a different animal in which the Brp blobs are closer to the GF dendrite so that their elimination greatly reduces the amount of spurious Brp-GF overlap.
(TIF)

S2 Fig Electron microscopy of JON-GF connection. (A) Sample electron microscope section from the publicly available dataset of Tobin et al, (2017) [36]. Section number 566, location x88624, y73927, zoom level 1. The GF dendrite (GF) is colored pale yellow, areas of close apposition with JO-A axons (A) delineated with a magenta line, and chemical synapse AZ colored green. (B) The inset shows a higher magnification view without false color, and the AZ indicated with a green arrow. The resolution (4 nm per pixel) is not sufficiently high enough to unequivocally identify gap junctions in the area of close apposition, although there are similarities with the figures in [9].
(TIF)

S1 File Data file for quantification of Brp overlap and GF branching. (XLSX)

S2 File Data file for quantification of Brp and ShakB immunostaining. (XLSX)
