# Supplementary figures and images for: Shaking-B misexpression increases the formation of gap junctions but not chemical synapses between auditory sensory neurons and the giant fiber of Drosophila melanogaster
Source: PLoS One. 2018 Aug 17;13(8):e0198710. doi: 10.1371/journal.pone.0198710 (PMC6097648; doi:10.1371/journal.pone.0198710)

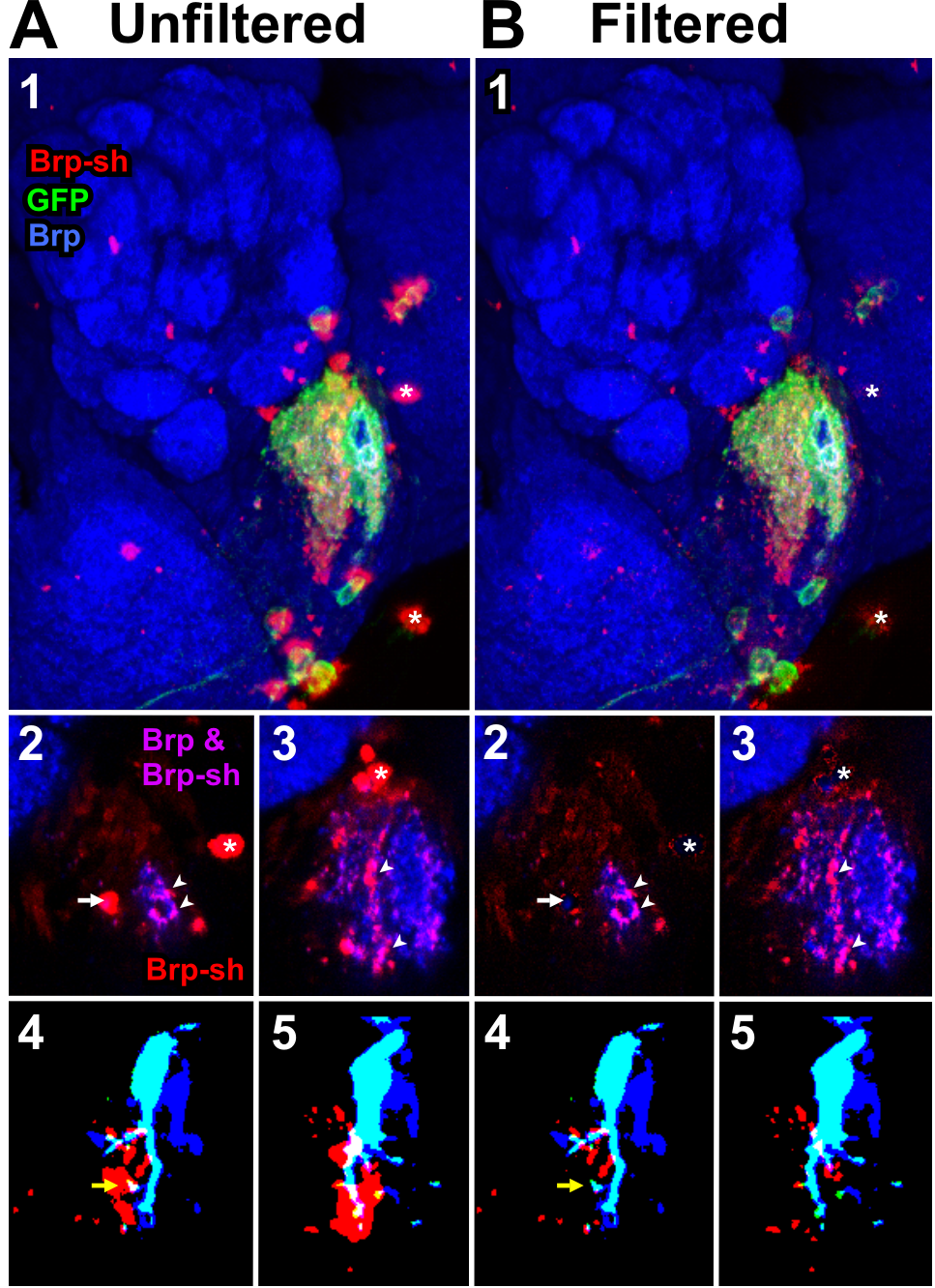

Supplement: S1 Fig — The A column shows unfiltered images, the B column shows images with the red channel filtered to remove large agglomerations. Panels 1–3 show a preparation in which JO15-GAL4 was used to drive UAS-brp-sh-strawberry (red: Brp-sh) and also UAS-CD8::GFP (green: GFP) in the A and B JONs. Native Brp protein was stained with nc82 antibody (blue: Brp). Panel1 is a maximum intensity Z-series projection through the length of the JON afferents, panels 2 and 3 are single slice views taken at the anterior tip and midway along the arbors, respectively. In these the GFP channel is removed for clarity. (A) Unfiltered images, showing large blobs that do not stain with nc82 (asterisks), some of which are associated with JO15-labeled cell bodies or axons. (B) Filtered images showing the almost complete removal of the artefactual blobs, leaving the nc82-stained Brp-sh puncta unaffected (arrowheads in 2 and 3). In panels 4 and 5 are shown preparations in which the GF axon was injected with NB (blue) and DA488 (green), making it cyan in color. Single sections are taken from the posterior region of the GF arbor. Panel 4 shows the same preparation and section as in Fig 2C3 –in this case filtering out the large blobs has little effect on the amount of overlap of Brp with the GF (white regions), although one contact region is greatly reduced (yellow arrow). In contrast, panel 5 shows the same region of a different animal in which the Brp blobs are closer to the GF dendrite so that their elimination greatly reduces the amount of spurious Brp-GF overlap. (TIF) [file pone.0198710.s001.tif]

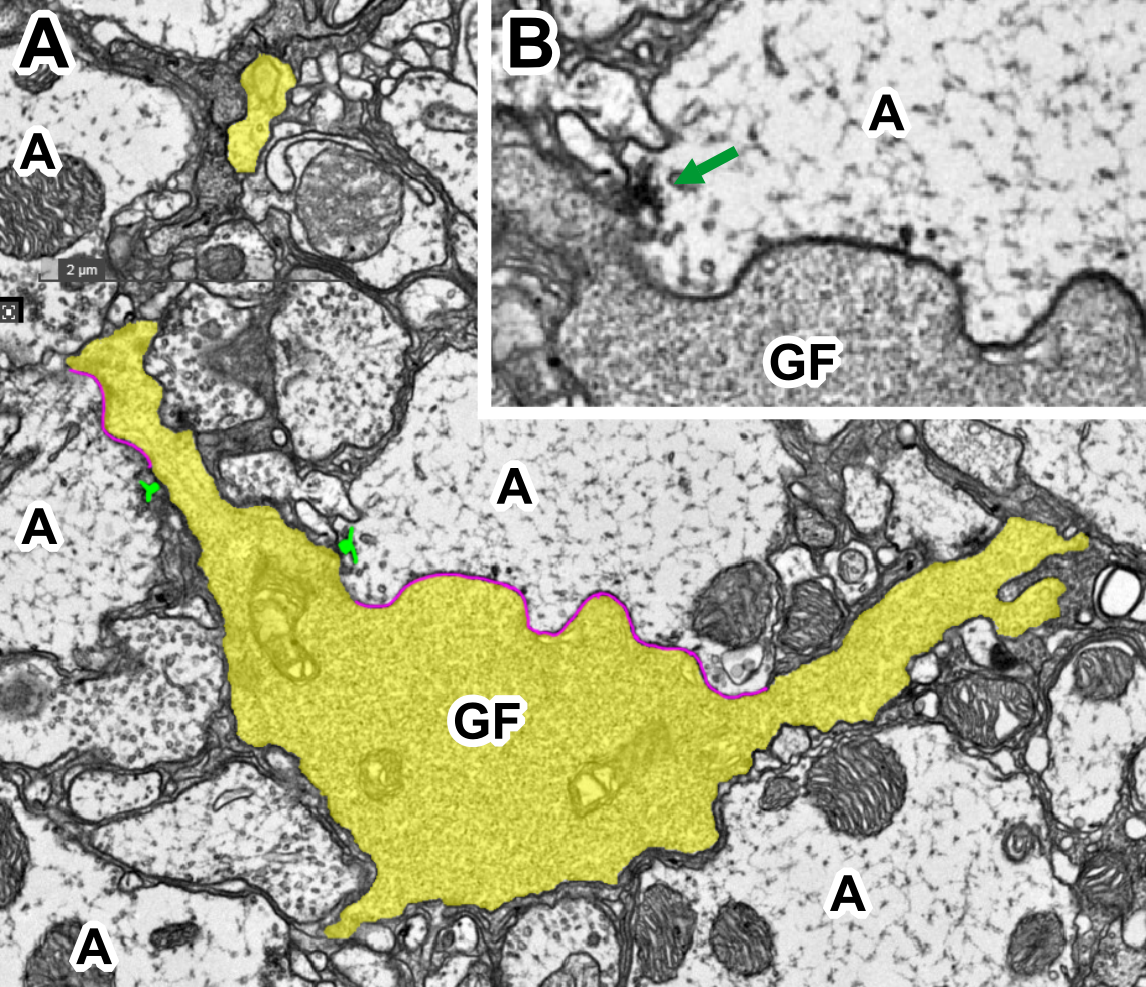

Supplement: S2 Fig — (A) Sample electron microscope section from the publicly available dataset of Tobin et al, (2017) [36]. Section number 566, location x88624, y73927, zoom level 1. The GF dendrite (GF) is colored pale yellow, areas of close apposition with JO-A axons (A) delineated with a magenta line, and chemical synapse AZ colored green. (B) The inset shows a higher magnification view without false color, and the AZ indicated with a green arrow. The resolution (4 nm per pixel) is not sufficiently high enough to unequivocally identify gap junctions in the area of close apposition, although there are similarities with the figures in [9]. (TIF) [file pone.0198710.s002.tif]
